# Growth faltering is associated with altered brain functional connectivity and cognitive outcomes in urban Bangladeshi children exposed to early adversity

**DOI:** 10.1186/s12916-019-1431-5

**Published:** 2019-11-25

**Authors:** Wanze Xie, Sarah K. G. Jensen, Mark Wade, Swapna Kumar, Alissa Westerlund, Shahria H. Kakon, Rashidul Haque, William A. Petri, Charles A. Nelson

**Affiliations:** 1 Labs of Cognitive Neuroscience, Division of Developmental Medicine, Boston Children’s Hospital, Boston, USA; 2 Harvard Medical School, Boston, USA; 3 Department of Applied Psychology and Human Development, University of Toronto, Toronto, USA; 4 ICDDR, B, Dhaka, Bangladesh; 5 Infectious Diseases & International Health, University of Virginia, Charlottesville, USA; 6 Harvard Graduate School of Education, Cambridge, USA

**Keywords:** Early adversity, Stunting, Malnutrition, EEG functional connectivity, Low-income countries

## Abstract

**Background:**

Stunting affects more than 161 million children worldwide and can compromise cognitive development beginning early in childhood. There is a paucity of research using neuroimaging tools in conjunction with sensitive behavioral assays in low-income settings, which has hindered researchers’ ability to explain how stunting impacts brain and behavioral development. We employed high-density EEG to examine associations among children’s physical growth, brain functional connectivity (FC), and cognitive development.

**Methods:**

We recruited participants from an urban impoverished neighborhood in Dhaka, Bangladesh. One infant cohort consisted of 92 infants whose height (length) was measured at 3, 4.5, and 6 months; EEG data were collected at 6 months; and cognitive outcomes were assessed using the Mullen Scales of Early Learning at 27 months. A second, older cohort consisted of 118 children whose height was measured at 24, 30, and 36 months; EEG data were collected at 36 months; and Intelligence Quotient (IQ) scores were assessed at 48 months. Height-for-age (HAZ) *z*-scores were calculated based on the World Health Organization standard. EEG FC in different frequency bands was calculated in the cortical source space. Linear regression and longitudinal path analysis were conducted to test the associations between variables, as well as the indirect effect of child growth on cognitive outcomes via brain FC.

**Results:**

In the older cohort, we found that HAZ was negatively related to brain FC in the theta and beta frequency bands, which in turn was negatively related to children’s IQ score at 48 months. Longitudinal path analysis showed an indirect effect of HAZ on children’s IQ via brain FC in both the theta and beta bands. There were no associations between HAZ and brain FC or cognitive outcomes in the infant cohort.

**Conclusions:**

The association observed between child growth and brain FC may reflect a broad deleterious effect of malnutrition on children’s brain development. The mediation effect of FC on the relation between child growth and later IQ provides the first evidence suggesting that brain FC may serve as a neural pathway by which biological adversity impacts cognitive development.

## Background

Exposure to adverse conditions in early childhood has been shown to exert both proximal and distal effects on physical and psychological health and development. Stunting is regarded as a primary indicator of chronic malnutrition—a serious biological hazard with long-term developmental consequences—and affects a large number of children worldwide, especially in low-income countries. According to recent reports by UNICEF, World Health Organization (WHO), and the World Bank Group, 159 million children under age 5 years can be classified as stunted (i.e., having a standardized height-for-age [HAZ] score that is 2 standard deviations [SDs] below the median of the WHO reference). Chronic malnutrition resulting in stunting has previously been associated with delayed brain development and poor cognitive performance [1, 2], which in turn has a profound impact on the degree to which children can achieve their developmental potential [3]. Although it is well known that sufficient nutrients are necessary for brain and cognitive development [4], the mechanistic pathways by which malnutrition in early childhood relate to later cognitive outcomes remain unclear. This is, in part, attributable to a scarcity of research using both neuroimaging and sensitive behavioral measurement in low-income settings where growth faltering is most prevalent.

In the current study, we investigate associations between stunted growth as an indicator of chronic malnutrition and brain functioning in Bangladeshi children living in impoverished, low-resource neighborhoods. Specifically, we explore the association between HAZ and source-space EEG functional connectivity (FC), and whether brain FC mediates the relation between HAZ and cognitive functioning. EEG FC in different frequency bands has been shown to be a useful tool to investigate the development of the efficiency and organization of brain networks among typically developing children, as well as children exposed to early adversity [5–9]. Variation in FC is often attributed to changes in the organization and functioning of brain networks. Abnormal patterns of FC within certain circuits due to biological adversity have been linked to deficits in later cognitive performance [10, 11]. For example, the FC in neonates’ subcortical, salience, and dorsal attention networks is correlated with maternal inflammation during pregnancy and predicts children’s working memory performance at 2 years of age [10]. It is therefore plausible that communication between cortical areas through neural oscillations in different frequency bands represents one pathway that is disrupted by chronic malnutrition during early childhood which, in turn, may lead to deficits in cognitive outcomes.

A large body of evidence from both human and animal studies supports the association between nutritional deficiencies (e.g., stunted growth) and atypical patterns of brain development [1]. For example, evidence from human postmortem studies revealed that 3- to 4-month-old infants suffering from malnutrition (indicated by low weight-for-age) showed reduced dendrite growth compared to well-nourished infants in the primary motor cortex (i.e., precentral gyrus) [12]. In addition, adults exposed to prenatal famine have white matter (WM) hyperintensities over the entire cerebrum on structural MRI. Increased WM volume among those exposed to famine may be caused by an inadequate supply of nutrients early in life to sustain and replace catabolized myelin and gliosis after myelin loss [13]. Animal models support histological evidence from malnourished infants in that studies of rodents have found that undernutrition is associated with reduced density of synapses and neurons in neural tissue over the cortex and alterations in callosal (interhemispheric) connections, likely caused by reduced neuron proliferation and changes in myelination and synaptic pruning [14–16]. The neuronal and volumetric changes in the brain associated with undernutrition may lead to poor cognitive outcomes. A growing literature has demonstrated the adverse effects of stunting on children’s cognitive development that, in turn, is believed to contribute to worse educational and labor-market outcomes, including lower income and poorer productivity [17, 18]. In addition, faltered growth during infancy and childhood compared to later adolescence is more likely to cause negative long-term effects on adult health and capital [18, 19].

The first few postnatal years represent a period of rapid neural change [20] and a critical window during which experiences have strong effects on neural and cognitive development [21]. Children living in low-resource settings are often exposed to a variety of biological, psychosocial, and environmental adversities beginning early in life [22]. Given the critical knowledge gap concerning the neural pathways by which growth faltering in early childhood affects cognitive outcomes, it is important to examine the associations between HAZ, brain functioning, and cognitive outcomes in children living in low-resource environments with high rates of stunting.

The current study recruited two cohorts of infants and toddlers residing in an urban slum in Dhaka, Bangladesh. For the “infant cohort,” the length of the infants was measured at 3, 4.5, and 6 months, and resting-state (baseline) EEG data were collected at 6 months. For the older toddler cohort, the height of the toddlers was measured at 24, 30, and 36 months, and their EEG data were collected at 36 months. Brain FC between cortical regions was estimated after cortical source reconstruction of the scalp-level EEG, with the aim to reduce the effects of volume conduction on FC between electrodes [23]. We primarily focused on the global FC over the entire brain, while exploratory analyses were also conducted to examine whether FC within and between certain lobes (i.e., frontal, temporal, parietal, and occipital) varied as a function of adverse experiences. Brain FC in the theta, alpha, and beta frequency bands were examined because activation in these bands is commonly used to study the neural correlates of cognitive development in the area of attention, memory, and emotion processing in infants and young children [24]. We also explored connectivity in the gamma band since activity in this band has been associated with cognitive outcomes such as language development [25] and exposure to adversity in infancy [26]. Prospective cognitive outcomes in the current study were assessed at 27 months for the infant cohort and at 48 months for the toddler cohort.

Longitudinal path analysis was conducted to test the associations between HAZ, brain FC, and cognitive outcomes, and whether brain FC mediated the relation between HAZ and cognitive outcomes. In these analyses, socioeconomic status (SES) and family caregiving were included as covariates as they are associated with child development and may confound the relation between child growth and neurocognitive development [21, 27]. We additionally controlled for children’s head circumference because of previously shown associations with physical growth and cognitive development in children in low- and middle-income countries [28]. Given the global impact of undernutrition on brain functioning and anatomical development shown in the literature, we hypothesized that stunted growth would be prospectively associated with distinct patterns of brain FC between multiple regions and circuits in the brain (i.e., whole-brain or global FC). We further hypothesized that stunted growth would be prospectively associated with worse cognitive outcomes, and brain FC would mediate the link between child growth and cognitive functioning.

## Method

### Participants

The final infant sample consisted of 92 (40M/52F) children whose growth data were collected at 3, 4.5, and 6 months. The final toddler sample consisted of 118 (65M/53F) children whose growth data were collected at 24, 30, and 36 months. All infants and toddlers were born ≥ 34 gestational weeks, with no known history of neurological abnormalities or traumatic brain injury, genetic disorders, or visual or auditory delays or impairments. The two cohorts originally each included 130 infants (56M/74F) or toddlers (72M/58F). Our study population was recruited from an impoverished neighborhood (urban slum) in Dhaka, Bangladesh, which was similar to other slum-dwelling populations in Bangladesh that are characterized by challenging living conditions including high rates of malnutrition, illiteracy, unemployment, and low family income [29]. The average monthly household income for the two cohorts was $187 (SD = 119) and $154 (SD = 107), respectively. These incomes fall below the current poverty line ($1.9 per household member per day) defined by the World Bank, which equals $173 to $289 per household per month for families with 3 to 5 members. A total of 50 participants were excluded from the final samples because they had missing growth measures or EEG data (*N* = 34), or their EEG data were excluded due to insufficient (< 60s out of 120s) clean data (*N* = 16) after artifact rejection. The final samples were representative of the original datasets in terms of sex, SES, and growth measures.

Ethical approval for the study was obtained from research review and ethics review committees at the International Centre for Diarrheal Disease Research, Bangladesh, and Institutional Review Boards at Boston Children’s Hospital and was in accordance with local guidelines and regulations. We collected written consent from the parents of the children who participated in the study.

### Growth measures

The supine length of the infants and the height of the toddlers were measured to the nearest 0.1 cm using a calibrated digital scale. Length/height measurements were taken twice during each assessment, and the average of the two values was calculated and used for the analyses. Participants’ HAZ at each time point was standardized based on WHO standards. The HAZ scores obtained at different time points were highly correlated for the infant (*r*s > 0.72) and toddler cohorts (*r*s > 0.91) and were therefore averaged across the three time points for both cohorts to obtain a stable estimate of physical growth over time (i.e., 3 to 6 months for the infant cohort, and 24 to 36 months for the toddler cohort) and to minimize measurement error or missing data at a certain time point. Stunting was defined as a HAZ that was 2 SDs below the median of the WHO reference. The average HAZ between 3 and 6 months for the infant cohort was − 1.14 (SD = 0.84), and the prevalence of stunting was 16.30% (15/92). The average HAZ between 24 and 36 months for the toddler cohort was − 1.64 (SD = 0.91), and the prevalence of stunting was 33.06% (39/118; 33.06%).

The HAZ was analyzed as a continuous variable in the linear regression models and longitudinal path analysis. Given the high prevalence of stunting at 36 months, we also categorized the toddlers into three groups, i.e., stunted (*N* = 39), middle HAZ (*N* = 39), and high HAZ (*N* = 40) groups, to examine and demonstrate how stunted children might be different from non-stunted children in terms of brain FC and cognitive functioning.

### Cognitive assessment

Cognitive outcome of the infant cohort was assessed with the Mullen Scales of Early Learning (MSEL) at 27 months (*M* = 26.84, SD = 2.41) for 74 out of the 92 infants. The scores for four subscales (fine motor, visual reception, receptive language, and expressive language) were standardized and used to calculate a composite score reflecting global cognitive development.

The cognitive outcome of the toddler cohort was assessed with the Wechsler Preschool and Primary Scale of Intelligence (WPPSI-III) at 48 months (*M* = 48.46, SD = .20) for 112 out of the original 118 children, since children older than 3 years of age tended to demonstrate a ceiling effect on MSEL. The full-scale Intelligence Quotient (IQ) score, a reliable and representative measure of general intellectual functioning, was calculated.

The MSEL and WPPSI were administered by local research assistants and psychologists. The items in the two assessments were translated and culturally adapted through pre-pilot tests, such as re-ranking the questions according to the difficulties in the Bangladeshi context [30]. Test-retest reliability has been shown by previous studies using these culturally adapted questionnaires with local Bangladeshi children [31, 32]. However, neither the MSEL nor the WPPSI have been standardized based on Bangladesh-appropriate norms, and thus, the American norms were used, which hinders direct comparisons between the scores for Bangladeshi children and those living in Western countries.

### EEG data collection and processing

The EEG data were collected at 6 months (*M* = 6.09, SD = .13) for the infant cohort and at 36 months for the toddler cohort (*M* = 36.88, SD = .19). EEG was recorded from a 128-channel HydroCel Geodesic Sensor Net (HGSN) that was connected to a NetAmps 300 amplifier (Electrical Geodesic Inc., Eugene, OR) while children watched a screensaver with abstract shapes and soothing sounds for 2 min. EEG recordings were offline filtered with an eighth-order Butterworth band-pass (1–50 Hz) filter. The filtered data were then segmented into 1-s epochs and inspected for artifacts using absolute and stepwise algorithms, as well as independent component analysis for removing components related to eye movements, blinks, and focal activity (see Additional file 1: Supplemental Information for details).

### EEG FC analysis in the source space

The processing stream for the source-space FC analysis used in the current study is illustrated in Additional file 1: Figure S1 (also see [33]). Cortical source reconstruction was conducted for the scalp EEG data using realistic head models created for both cohorts using age-appropriate (i.e., 6 and 36 months) average MRI templates [34]. Distributed source reconstruction of the EEG time-series was conducted, and reconstructed source activities were segmented into 48 cortical regions of interest (ROIs) using the LPBA40 brain atlas [35]. Whole-brain FC between the 48 ROIs was estimated using the weighted phase lag index (wPLI [36]), a widely used measure of “phase-to-phase synchrony,” for different age-appropriate frequency bands: theta (6 months, 3–6 Hz; 36 months, 3–7 Hz), alpha (6 months, 6–9 Hz; 36 months, 7–10 Hz), beta (6 months, 10–20 Hz; 36 months, 11–20 Hz), and gamma (6 and 36 months, 20–40 Hz) bands [37, 38].

The 48 ROIs were further categorized into four different lobes—frontal (F), temporal (T), parietal (P), and occipital (O), and the FC within and between the four lobes (i.e., FF, FT, FP, FO, TT, TP, TO, PP, PO, OO) were calculated [39]. The list of the ROIs for the four lobes is included in Additional file 1: Supplemental Information. Exploratory analyses were conducted to examine the FC within and between the four lobes as a function of HAZ, with adjustment for multiple analyses with a false discovery rate (FDR) of 5%.

### Covariates

SES was assessed shortly after birth via home observations and standardized questionnaires. SES was defined and calculated as a latent factor based on multiple correlated indicators including income-to-needs quartiles, house construction materials, and family assets [30]. Family caregiving activities were also assessed at the time of the EEG assessment via maternal interviews using the Family Care Indicators (FCI) [40], which includes five subscales to assess home stimulation. Four of the five subscales assess the varieties of play materials, books, magazines, and newspapers that are available in the household. The other subscale “play activities” assesses the number of stimulating activities that the parents or other caregivers engaged in with the child within the last 3 days. The total score of these subscales was used as the index of family caregiving. Finally, head circumference was measured in centimeters at the time of the EEG data collection.

### Statistical analysis

Linear regression models were run in IBM SPSS Statistics (version 25, IBM Corp, Armonk, NY) to examine the association between HAZ and brain FC and between brain FC and cognitive functioning. The linear regression models were run for the FC in each frequency band separately. Only frequency bands that showed significant relations with HAZ and cognitive outcomes were further tested in the longitudinal path analysis linking HAZ to later cognitive functioning. The hypothesized mediation model (Fig. 5) was tested using longitudinal path analysis in Mplus (version 7.4). Specifically, the mediation model tested the indirect effect of HAZ on cognitive outcomes via brain FC. Missing values on cognitive outcomes (e.g., missing WPPSI scores, *n* = 6) were handled using full-information maximum likelihood (FIML) estimation with robust standard errors. Model fit was evaluated based on a non-significant *X*^2^ (*p* > 0.05), CFI > 0.95, SRMR < 0.08, and RMSEA < 0.06. Indirect effects were estimated using bootstrapping across 5000 draws with bias-corrected confidence intervals.

## Results

### Child growth and brain FC

**Fig. 1:**
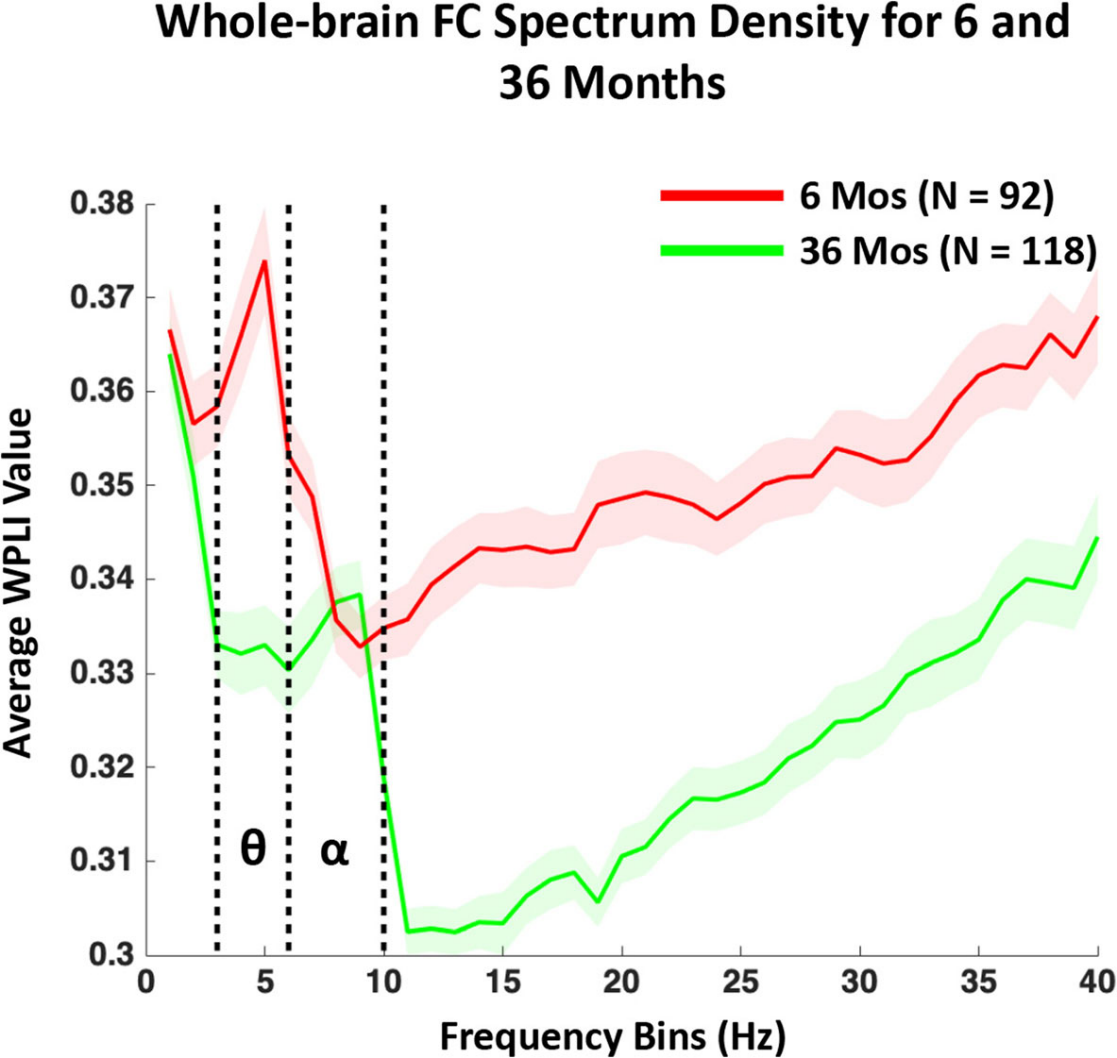
Average whole-brain functional connectivity “spectrum density” (FCSD) for the 6-month-old (red) and 36-month-old (green) cohorts. The shade area represents the standard errors across all the participants at each frequency bin. Note: The FCSD peaks in the theta (θ) and alpha (α) bands for the 6- and 36-month-old cohorts respectively

**Fig. 2:**
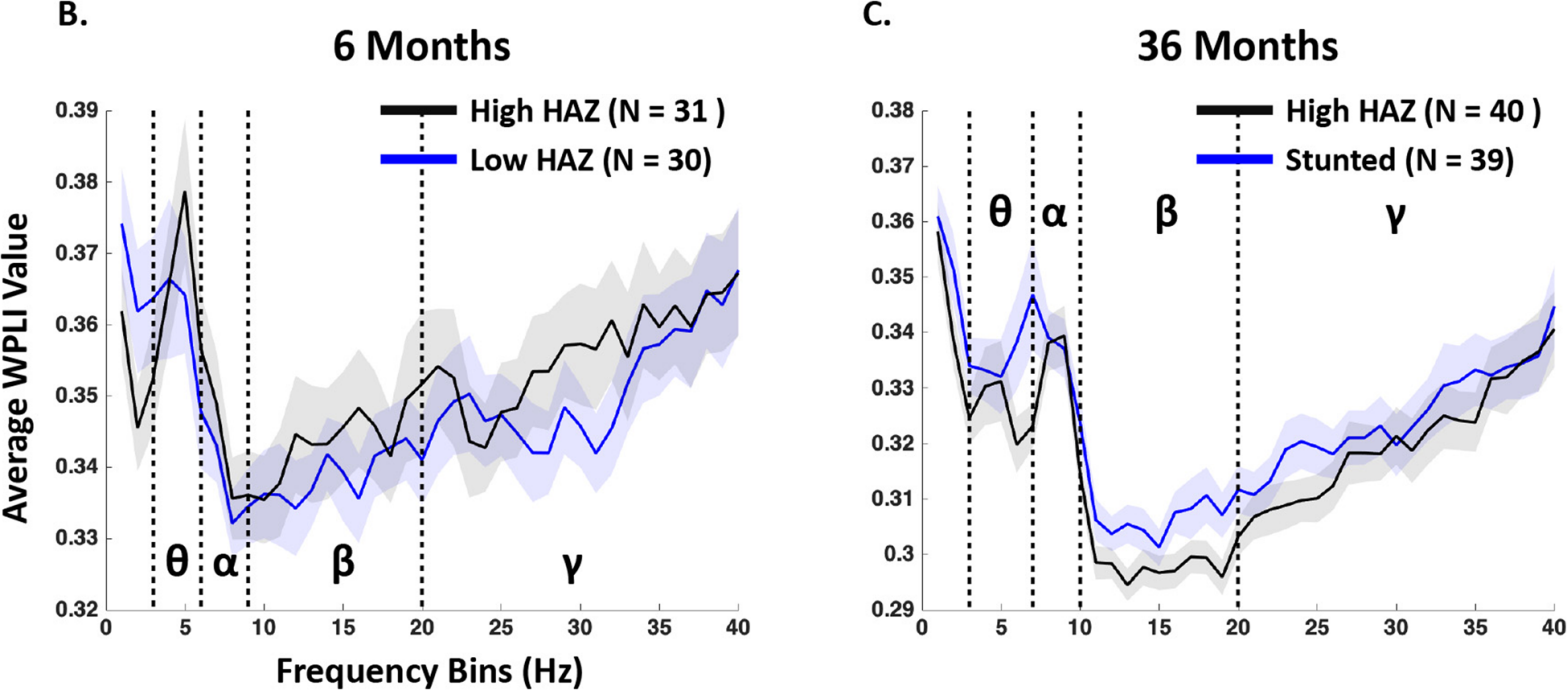
Whole-brain FCSD for the 6-month-old (**b**) and 36-month-old (**c**) cohorts across the theta (θ), alpha (α), beta (β), and gamma (γ) bands. For the 6-month cohort, the FCSDs for the top one third of the infants with the highest HAZ and the bottom one third with the lowest HAZ were plotted. For the 36-month cohort, the FCSDs for the top one third of the children (high HAZ) and the stunted children (one third of the cohort) were plotted. The shade areas represent the standard errors for each group

Average whole-brain FC was found to vary across frequency bands and peaked in the theta band (*M* = 5.68 Hz, SD = 1.23) for the 6-month-old infants and in the alpha band (*M* = 7.67, SD = 1.48) for the 36-month-old children (Fig. 1). The peak frequency picked up by the “findpeaks.m” function in MATLAB was analyzed as a function of HAZ for the two cohorts, and no significant associations were found, although the peak frequency of global FC at 36 months appeared to be slower (lower) for the stunted children (Fig. 2).

Linear regression models were performed to test the association between HAZ and FC in different frequency bands as well as other covariates. There was no association of HAZ or other covariates (SES, family caregiving, and head circumference) with whole-brain FC for the infant cohort in any of the frequency bands.

**Fig. 3:**
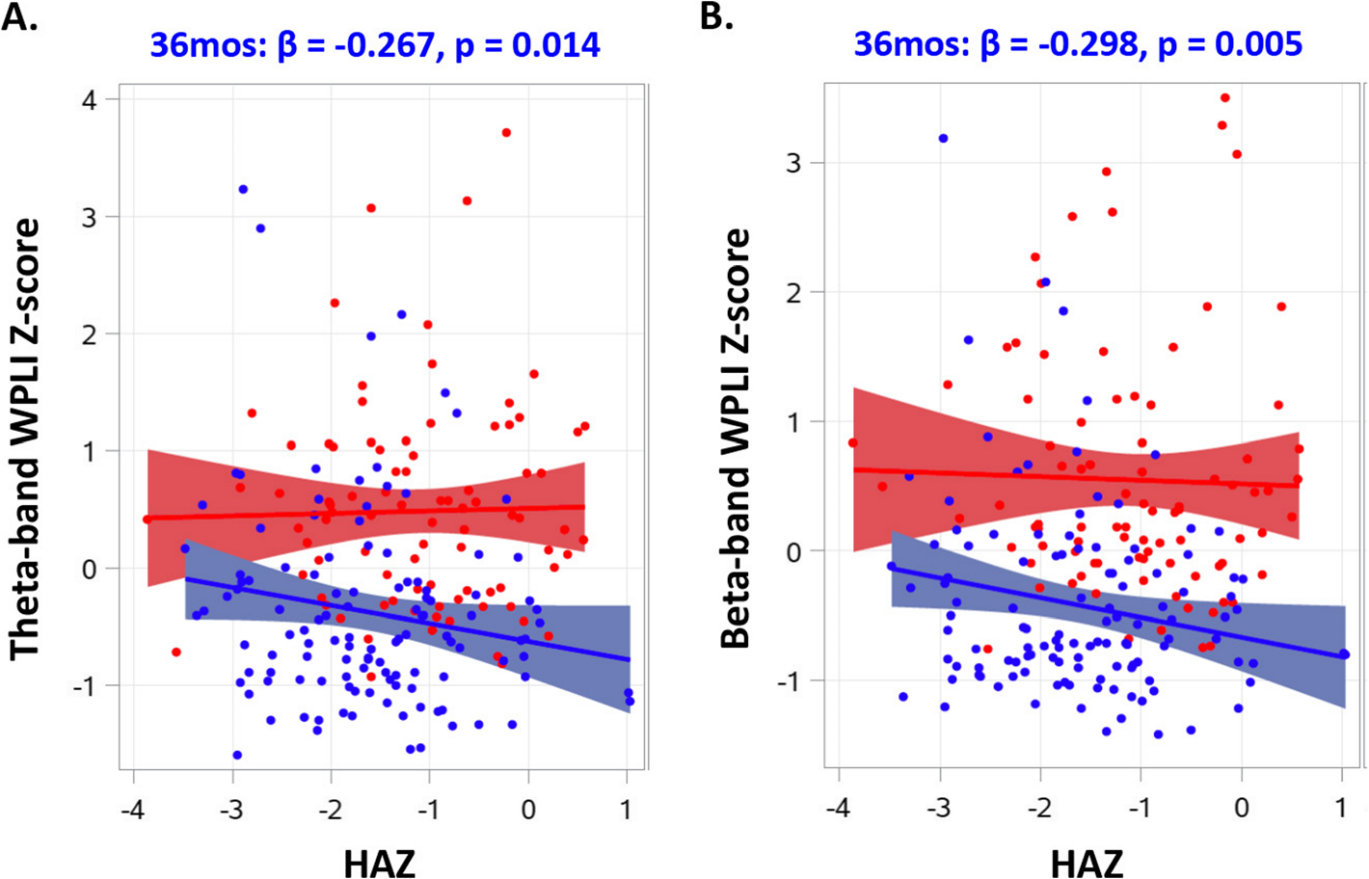
The linear association between HAZ and standardized brain FC values in the theta (**a**) and beta (**b**) bands. The regression lines are plotted separately for the 6-month-old (red) and 36-month-old (blue) children. The shade areas represent the 95% CI of the regression line

**Fig. 4:**
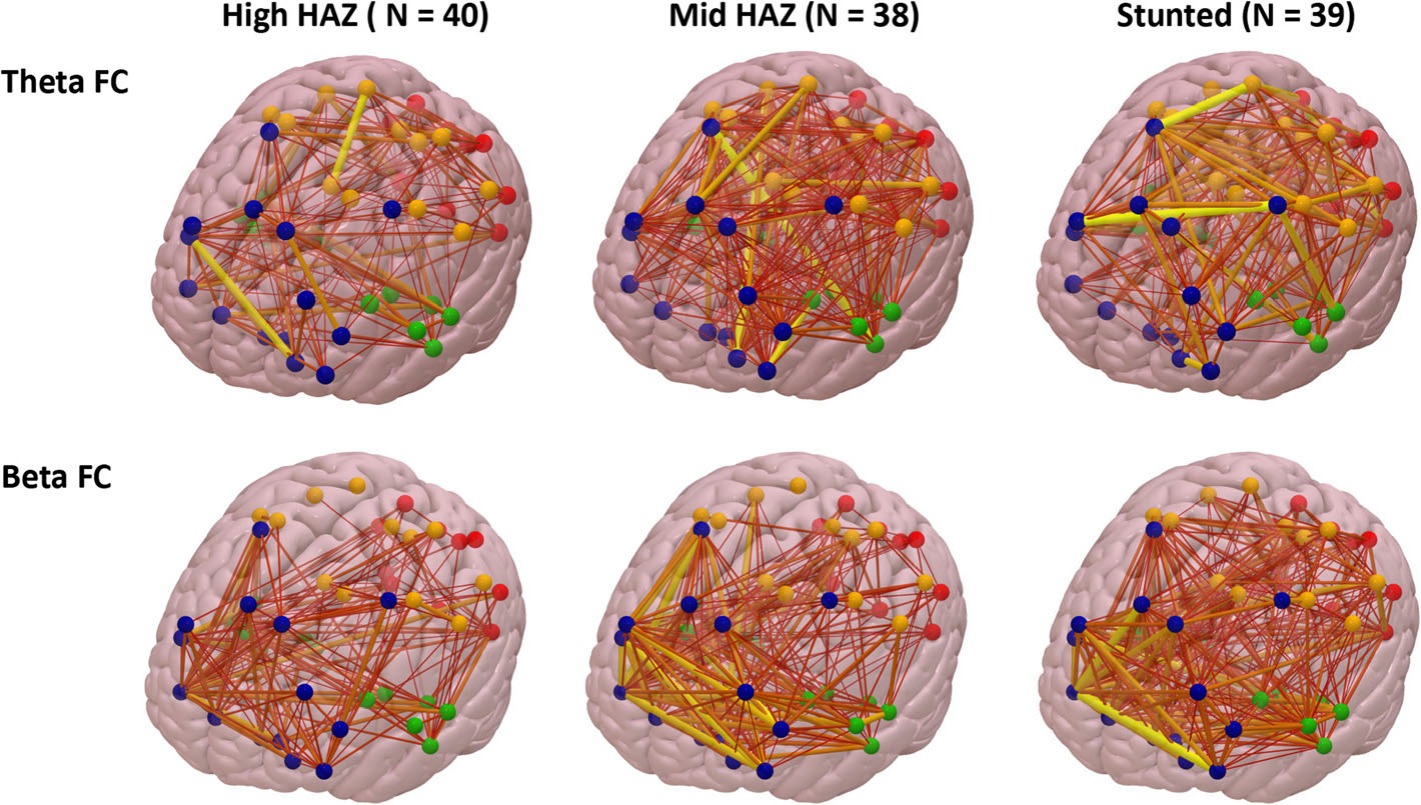
The average brain FC in the theta (top) and beta (bottom) bands is illustrated for stunted and non-stunted groups. The connections (edges) between cortical regions in the brains are plotted with the same threshold for all three groups, and thus, the weakest connections are not shown in the brains and the thicker the line is the higher the FC value is. Brain ROIs belonging to different lobes are in different colors—frontal ROIs in blue, temporal ROIs in green, central and parietal ROIs in yellow, and occipital ROIs in red. The corresponding adjacency matrices for each brain FC figure can be found in Additional file 1: Figure S3

For the toddler cohort, HAZ between 24 and 36 months was negatively correlated with FC at 36 months in the theta (*β* = −.267, *p* = .014) and beta bands (*β* = −.298, *p* = .005), such that children with lower HAZ showed stronger whole-brain FC (Fig. 3). The linear regression models also revealed associations that were close to significance for the FC in the beta band and SES (*β* = .190, *p* = .067). No associations were found involving FC in the gamma band. The 36-month-old children were further divided into stunted and non-stunted groups. Whole-brain FC in the theta and beta bands for the growth faltered children was stronger than that for the high HAZ group (Figs. 2 and 4; also see Additional file 1: Figure S3).

### Child growth and FC within/between brain lobes

Exploratory analyses were conducted to examine whether FC in the theta and beta bands within and between the four brain lobes varied as a function of HAZ. The same linear regression models tested in the previous section were run replacing whole-brain FC with FC within or between brain lobes (i.e., FF, FT, FP, FO, TT, TP, TO, PP, PO, OO), with FDR (5%) adjustment for the *p* values from multiple analyses. These analyses showed that, for the analyses focusing on the theta band, HAZ was negatively associated with FC within and between most of the brain lobes (adjusted *p*s < 0.05), except for FC between the temporal and parietal lobes and FC within the occipital lobe. In contrast, HAZ was only associated with beta-band FC between the occipital and temporal lobes and between the occipital and parietal lobes (adjusted *p*s < 0.05).

### Brain FC and later cognitive outcomes

Linear regression analyses were then performed to investigate whether whole-brain FC in the different frequency bands was prospectively associated with IQ. In the toddler cohort, we found significant negative associations between brain FC at 36 months in the alpha (*β* = −.202, *p* = .020) and beta (*β* = −.179, *p* = .042) bands and IQ scores at 48 months. The association between theta-band FC at 36 months and IQ at 48 months was marginally significant, *β* = −.169, *p* = .053. In addition, the linear regression models revealed a significant positive association between SES and IQ (*β* = .320, *p* = .001). In contrast, no associations were found between brain FC MSEL composite scores in the infant cohort.

### Longitudinal path model linking child growth to cognitive outcomes via brain FC

**Fig. 5:**
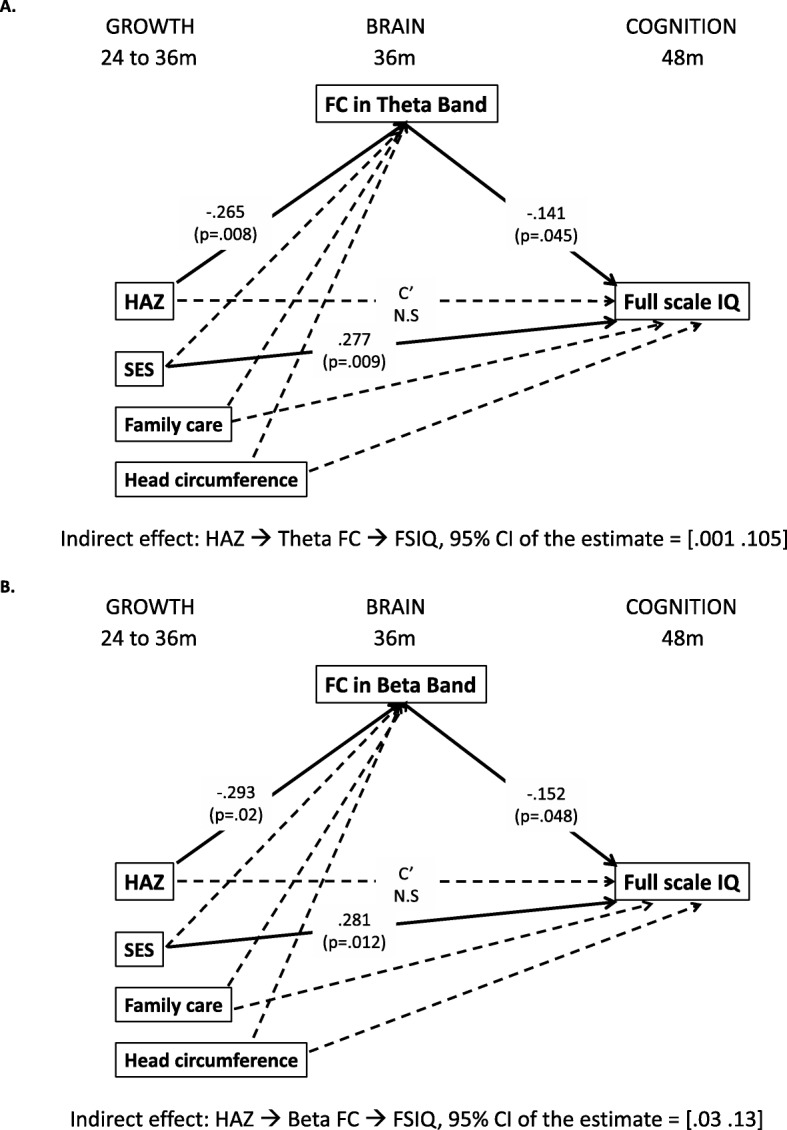
Multivariate mediation models tested with longitudinal path analysis: **a** the model with the theta-band FC and **b** the model with the beta-band FC. The numbers in parentheses are the *p* values, and the numbers above the *p* values are the standardized estimates. Solid lines represent significant associations, while dashed lines represent non-significant associations

Mediation models were tested only for the toddler cohort given that this was the cohort for whom the strongest associations between HAZ, brain FC, and cognitive functioning were observed. We tested the indirect effect of HAZ on IQ scores at 48 months via brain FC in both the theta and beta bands separately (Fig. 5a, b). The “theta model” showed acceptable model fit: *χ*^2^(1) = .277, *p* = .599; CFI = 1.0; SRMR = .009; RMSEA < .001; however, the “beta model” showed poorer model fit as indicated by smaller CFI and higher RMSEA values than what is typically used to define acceptable model fit: *χ*^2^(1) = 2.918, *p* = .088; CFI = .939; SRMR = .030; RMSEA = .127. The mediation model was not run for FC in the alpha and gamma bands because they were not associated with HAZ.

The longitudinal path analysis revealed that HAZ was negatively associated with brain FC in the theta and beta bands at 36 months, which in turn were both negatively associated with IQ at 48 months (Fig. 5). The direct effect of HAZ on IQ was not significant in the path model including all the covariates, although stunted children did show lower IQ compared to non-stunted children (Additional file 1: Figure S2). Significant indirect effects of HAZ on IQ via brain FC in the theta band (95% CI of the standardized estimate [.001, .105]) and beta band (95% CI of the standardized estimate [.03, .13]) were observed (Fig. 5a, b).

## Discussion

The current study provides the first evidence that physical growth early in life is associated with variations in network connectivity in the brain as inferred from theta- and beta-band FC between brain regions among children living in a low-income country. In a cohort of toddlers, we also show that brain FC is, in turn, prospectively associated with later cognitive performance and that there is an indirect effect of HAZ measured between 24 and 36 months on IQ measured at 48 months via FC at 36 months. In contrast, in a younger cohort of infants, no association was found between HAZ measured between 3 and 6 months and brain FC at 6 months or MSEL composite score at 27 months.

The associations between HAZ and whole-brain FC for the toddler cohort may reflect a broad deleterious effect of growth faltering on children’s brain functioning. The neural oscillations in the theta and beta bands have been associated with cognitive functions such as sustained attention, executive attention, and working memory [37, 41–43]. Atypical patterns of FC in these frequency bands may therefore be associated with deficits in cognitive functioning in a number of critical domains. Interestingly, the pattern of associations between HAZ and FC in these two frequency bands was found to be slightly different, such that stunting was associated with stronger FC in the theta band for most of the brain regions, especially connections involving the frontal lobe, whereas the association between HAZ and FC in the beta band was more prominent for connections involving the occipital lobe. This discrepancy could be due to differences in the location of the cortical sources of theta and beta rhythms in early childhood.

One plausible explanation for the heightened FC among children with lower HAZ (including stunted children) is under/delayed synaptic pruning due to malnutrition, in turn leading to the failure to eliminate unnecessary connections as well as less organized and more superfluous pathways between brain networks [13, 44]. Children’s experience plays a key role in synaptic pruning, which begins in the first year after birth and continues through adolescence [45]. Delayed synaptic pruning may occur in stunted children due to a lack of stimulation and input from the environment. A second explanation of the negative association between HAZ and brain FC is that higher connectivity may reflect a more adaptive or compensatory neural response to make up for delayed brain anatomical development in growth faltered children. For instance, higher connectivity could indicate less efficiency of neural communications between cortical regions, which requires more effort for children who are stunted. This second explanation is consistent with findings from a recent fMRI study which demonstrated that maternal inflammation level (IL-6 concentration) is positively associated with functional connectivity in some brain networks and better developmental outcomes [46]. Indeed, the authors of that study suggested that increased FC may reflect an adaptive neurodevelopmental response to inflammation exposure. Finally, a third possibility is that greater functional connectivity across the brain may reflect less network segregation (differentiation) between functional submodules and a less mature connectivity pattern, which is more similar to the network organization found in younger infants [47]. Future research that makes use of sophisticated techniques such as graph theory will be useful to elucidate the likelihood of this hypothesis [6].

The current study also showed that global brain FC decreases from 6 to 36 months and the peak FC frequency increases with age (Fig. 1). This finding provides insights into the developmental course of EEG brain FC—in this case “phase-to-phase synchrony”—in early childhood. The increase of the peak FC frequency from 6 to 36 months is consistent with the known changes in EEG power in childhood, which could reflect typical neural maturational processes [48]. As noted above, the general decrease of brain FC with age may reflect increased synaptic pruning occurring in childhood. If this is the typical pattern of EEG FC over the course of childhood, higher FC among stunted children may suggest delayed brain development. Future research should continue to investigate whether changes in brain FC during childhood follow a linear or non-linear trajectory by including more age groups between 6 and 36 months and using different functional connectivity methods (see the results with “imaginary part of the coherency (iCOH)” in Additional file 1: Supplemental Information).

The absence of an association between HAZ and FC in the alpha band may be due to the fact that synchronization in this band was suppressed during the experiment when children were watching (attending to) a screensaver with abstract shapes, as alpha is known to be attenuated while engaging in attention-demanding tasks. A second possibility is that using a priori defined boundaries might overlook the most prominent alpha-band FC, as the individual alpha peak frequency changes with age over childhood (Fig. 1; also see [49]). Although additional analysis with FC defined with individual alpha peaks failed to show a significant association between alpha-band FC and HAZ (Additional file 1: Supplemental Information), future research may consider using individually defined boundaries to measure alpha-band power and FC in children.

A particularly compelling finding of the current study is that the association between child growth and developmental quotient (i.e., IQ) operated through brain FC in the theta and beta bands in the toddler cohort. Biological adversity related to inflammation has recently been shown to impact FC and cognitive abilities in studies using fMRI techniques [10, 46]. Our finding adds to this growing literature by showing that FC mediates the relation between biological adversity vis-à-vis growth stunting and later cognition, such that FC may serve as a neural pathway by which exposure to early adversity (including malnutrition) affects cognitive outcomes. As a practical matter, the current study suggests that EEG—a measure that is less expensive and easier to implement than MRI—has the sensitivity to detect differences in brain FC related to early adversity and later cognitive functioning in low-resource settings.

The high prevalence of stunting in the 36-month cohort highlights the severity of deficits in neurobiological development in this sample of Bangladeshi children. In this cohort, 33.1% of the children were classified as stunted. This prevalence rate is comparable to a report by the Bangladesh Demographic and Health Survey in 2014, which showed that the prevalence of stunting is 33.5% among children under age 15. In the current study, the prevalence of growth faltering at 6 months was relatively lower than that at 36 months, with approximately 16% of the infant cohort meeting criteria for stunting. The increased prevalence of stunting from 6 to 36 months is consistent with the standard trajectory of children with poor nutrition, such that children with poor early nutrition fall off the growth trajectories across early childhood as they age [50]. Moreover, consistent with the robust literature suggesting the role of SES in cognitive development [27, 51], the current study showed that SES was positively associated with IQ. Given a large number of variables that covary with child growth, researchers should be mindful that deficits in brain and cognitive development are likely caused by a constellation of interacting factors for children living in low-income countries [52].

The absence of an association between growth faltering and FC or cognitive outcomes in the 6-month infant cohort suggests that the impact of growth faltering on brain and cognitive development may build over time and either (a) not be detected in the first half year of life or (b) this association may not yet be present. This explanation is supported by a recent study reporting very small effect sizes in the association of growth measures during infancy and cognitive development among children living in low-income countries [28]. Alternatively, relatively lower variance in HAZ within the infant compared to toddler cohort (including fewer stunted children) may have reduced the ability to detect significant associations between HAZ and FC. Another possibility is that breastfeeding, which was prominent during the first 6 months, protected children in the infant cohort from growth faltering, or from the effects of growth faltering on brain and cognitive development [50]. The current findings for the infant cohort highlight the need for future research to follow these children longitudinally and monitor their growth trajectories throughout childhood.

### Limitations

One limitation of the current study is the usage of an arbitrary sparsity threshold of 0.2 in the FC analysis. Although we have found similar results by using other thresholds (e.g., 0.1 and 0.3; Additional file 1: Supplemental Information), future research may consider measuring the area under the curve with multi-thresholds and permutation correction to overcome the threshold bias [53].

A second limitation of the current study is that we used HAZ as a proxy for malnutrition, but it is conceivable that malnutrition may have a direct effect on brain FC that is independent of HAZ. It is therefore possible that the deleterious effects of malnutrition are not fully captured by HAZ. It is also possible that HAZ is capturing more than just malnutrition risk. For example, HAZ may correlate with multiple co-acting factors related to severe adversity, such as SES, sanitation, inflammation, and parental stress, all of which may impact brain FC and cognition [54]. Thus, future studies that have direct measurement of nutritional levels will be important to determine if the results reported herein are robust and indeed reflect individual differences in FC and cognition as a function of malnutrition.

A third limitation is that our study might be underpowered to tease apart the variance explained by all the factors included in the statistical models. This could explain some of the unexpected non-significant effects, such as the non-significant association of family caregiving and brain FC and cognitive outcomes for the toddler cohort. Data sharing and collaboration between multiple sites is of central importance to facilitate a more thorough examination of the contribution of different adverse experiences to child brain and cognitive development in low-income countries.

## Conclusion

The current study is the first to investigate the relation between growth faltering, brain FC, and cognitive outcomes in children living in a low-income country. Our findings indicate that growth faltering, an indicator of chronic malnutrition, is prospectively associated with exaggerated EEG functional connections in the brain, which in turn are prospectively associated with poorer cognitive outcomes in the first 5 years of life. Findings from the current study advance our understanding of the neural pathways by which faltered growth could be associated with cognitive development, and this advancement may have a substantial impact on developing efficient interventions for children living in low-income countries.

## Supplementary information

**Additional file 1.** This file provides supplemental information on the methods and results of the current study, as well as a figure demonstrating the pipeline for source-space EEG functional connectivity analysis.

## Data Availability

The EEG dataset has been published in BIDS format and is available to be downloaded: Xie, Wanze (2019), “CRYPTO and PROVIDE EEG Data_Infants and Children”, Mendeley Data, V2, 10.17632/xc9vr5s52g.2.
