# Supplementary material for: Growth faltering is associated with altered brain functional connectivity and cognitive outcomes in urban Bangladeshi children exposed to early adversity
Source: BMC Med. 2019 Nov 25;17:199. doi: 10.1186/s12916-019-1431-5 (PMC6876085; doi:10.1186/s12916-019-1431-5)
Supplement: Supplementary file 1 — Additional file 1. This file provides supplemental information on the methods and results of the current study, as well as a figure demonstrating the pipeline for source-space EEG functional connectivity analysis. [file 12916_2019_1431_MOESM1_ESM.docx]

**Supplemental Information**

This document provides supplemental information on the methods and results of the current study.

**Method**

*EEG Data Collection and Processing*

EEG was recorded from a 128-channel HydroCel Geodesic Sensor Net (HGSN) that was connected to a NetAmps 300 amplifier (Electrical Geodesic Inc., Eugene, OR) while children watched a screensaver with abstract shapes and soothing sounds for 2 mins. The EEG recording was referenced online to a single vertex electrode. Channel impedance was kept below 100 kΩ and signals were sampled at 500 Hz.

EEG recordings were preprocessed using EEGLAB[1] and ERPLAB[2] toolboxes in MATLAB (R2017a, the Mathworks, Inc.). The continuous EEG data were filtered with an 8^th^ order Butterworth band-pass filter with a pass band of 1 – 50 Hz. The low-pass cut-off was set to be below the gamma band to reduce the effect of the muscle- and movement-related artifacts on the children EEG data. The filtered data was then segmented into 1s epochs. The EEG epochs were inspected for artifacts using both absolute and stepwise algorithms (EEG > 100 μV or EEG < -100 μV). Channel interpolation was conducted using a spherical spline interpolation with the EEGLAB function “eeg_interp” if there were fewer than 18 (15%) electrodes that were missing or had bad data[3, 4]. Independent component analysis (ICA) was also conducted to remove components related to eye movements, blinks, and focal activity. The functions and algorithms in SASICA[5] and ADJUST[6] were used to identify artificial components, and only those marked by both toolboxes were removed from the data. Each child must have at least 60 clean epochs (50%) to be included for further analyses. The number of artificial ICA components and the number of epochs included in the final analysis did not change as a function of the growth measures, *r*s < .1, *p*s >= .29. For the participants included in the final samples, the mean numbers of clean epochs were 103.59 (SD = 16.64) and 113.0 (SD = 12.05) for the infant (N = 92) and toddler (N = 118) cohorts respectively. The number of clean epochs was not correlated with the growth measures for the two cohorts, *r* = -.038, *p* = .73 and *r* = .066, *p* = .48.

*EEG Functional Connectivity (FC) Analysis in the Source Space*

The pipeline for the source-space FC analysis used in the current study was illustrated in Supplemental Figure 1 (also see[7]). Cortical source reconstruction was conducted for the scalp EEG data with the Fieldtrip toolbox[8]. Realistic head models were created for both 6- and 36-month-old cohorts using age-appropriate average MRI templates selected from the Neurodevelopmental MRI Database[9, 10]. Anatomical MRI templates were segmented into component materials, and a forward model was created for each age group using the Finite Element Method (FEM) with the gray matter being used as source volumes (5 mm grids). Age-appropriate skull conductivity values (0.066 Ω∙m^-1^ for the 6-month model and 0.036 Ω∙m^-1^ for the 36-month model) were used to build the models[11]. The forward model was then used to estimate the lead field matrix and the spatial filter matrix, i.e., the inverse of the lead field matrix.

Distributed source reconstruction of the EEG time-series was conducted with the exact-LORETA (eLORETA[12]) as the constraint for inverse modeling. The source volumes were segmented into 48 cortical regions of interest (ROIs) using the LPBA40 brain atlas[13]. The reconstructed time-series in the source volumes surrounding the centroid of each ROI were averaged to represent the source activation for each ROI[14].

FC analysis was conducted with the source-space time-series for the 48 cortical ROIs. Given the dramatic changes in the peak frequency of different frequency rhythms in the first few years of life (Marshall et al., 2002; Perone et al., 2018), age-appropriate frequency bands were used for the theta (6 mos: 3 – 6 Hz; 36 mos: 3 – 7 Hz), alpha (6 mos: 6 – 9 Hz; 36 mos: 7 – 10 Hz), beta (6 mos: 10 – 20 Hz; 36 mos: 11 – 20 Hz) and gamma (6 and 36 mos: 20 – 40 Hz) rhythms.

The FC between ROIs in different frequency bands was estimated with weighted phase lag index (wPLI[15]), a measure that weights the phase differences according to the magnitude of the leads and lags so that phase differences close to zero generated by noise perturbations would only have a marginal contribution to the results. In addition, random permutation of trials was applied to get rid of the effect of number of observations (trials) on the wPLI estimation[15], as the number of trials did differ between individuals. In specific, 60 trials were randomly selected from all the trials (varying between 60 and 120) to calculate the wPLI value between ROIs. This procedure was repeated for 50 times and the average wPLI value was calculated. FC analysis resulted in 48 x 48 weighted adjacency matrices, with each element in the matrix representing the connectivity between a pair of ROIs. The Fisher’s r-to-z transformation was applied to the values in the matrices to improve the normality of their distribution. A sparsity threshold of .2 (20%) was applied to the matrices to retain the strong and eliminate the weak or noise connections, as well as to keep the same number of connections across matrices[16]. Analyses were also done with thresholds of .3 and .1 to exclude the possibility that the results of the experiment are driven by the choice of network thresholds, and similar results were obtained across thresholds.

**Results**

*Validation Analysis of EEG Functional Connectivity (FC) Estimated with iCOH*

The estimation of EEG FC could be affected by the algorithms or methods used to calculate the phase-based relationship between two time series[17]. Therefore, we further estimated brain FC using the imaginary part of the coherency (iCOH[18]). The correlation between HAZ and FC estimated with iCOH was calculated to validate the results from using the wPLI method presented in the results section of the manuscript. The analysis with FC estimated with iCOH yielded very similar results to those with wPLI, i.e., there were negative correlations between the growth measures and brain FC in the theta and beta bands for the 36-month-old cohort (r = -.25, p = .015; r = -.28, p = .0026).

*Additional Analysis of EEG FC and HAZ*

There were two 3 years old children having average HAZ values greater than 1 (Figure 3), which is not very common for children living in such impoverished neighborhoods. Thus, we conducted additional sensitivity analyses to examine if the association between HAZ and FC in theta and beta bands remained the same after excluding these two participants. Results showed that HAZ was still negatively associated with FC in the theta (β = -.226, *p* = .034) and beta (β = -.274, *p* = .009) bands.

Additional regression analyses were also performed to examine the association between HAZ at 36 months and concurrent EEG FC in the theta and beta bands. Results showed that HAZ at 36 months was negatively associated with FC in theta (β = -.311, p = .005) and beta (β = -.319, p = .003) bands. These findings were comparable to those using averaged HAZ across the three measures between 24 and 36 months.

*Alpha-band FC Defined with Individual Alpha Peaks*

The absence of an association between alpha-band FC and HAZ might be due to the usage of a priori defined boundaries to measure the alpha-band FC. It is likely that the most prominent alpha FC did not fall within the predefined band for some individuals, as the alpha peak frequency changes with age over childhood (Figure 1). Therefore, we conducted additional analysis to examine the association between HAZ and alpha-band FC measured with individually defined peak frequency.

Individual alpha-band FC was first computed with the FC value at the peak FC frequency identified for each participant. Individual alpha peak frequency was defined as the frequency bin showing the maximum FC in the “broad alpha band” (4 – 9 Hz for infants; 4 – 11 Hz for toddlers) using the “findpeaks.m” function in MATLAB. There were four infants did not show a peak, and thus the FC at 6 Hz was used as their alpha-band FC. Correlation analysis showed that alpha-band FC at the peak frequency was highly correlated with the average alpha-band FC for the toddler cohort (r = .795, p < .001), but this correlation was not significant for the infant cohort (r = -.11, p = .335). Regression analysis showed that peak alpha-band FC was not associated with HAZ for either the infant (β = .087, p = .435) or the toddler (β = -.114, p = .299) cohort, which is consistent with the results found with average alpha-band FC.

We further measured the alpha-band FC using individual peak frequency identified with EEG power spectrum density (PSD). Global log-transformed PSD (i.e., averaged across all electrodes) was calculated for each participant, and the 1/f trend of the PSD was then modeled and subtracted from the data (Dickinson et al., 2018). Individual alpha peak frequency was identified as the frequency bin with the maximum power in the broad alpha band. The results showed that PSD at 6 months peaked at 5.70 Hz (SD = .912), which is comparable to the FC peak frequency. However, there were only 27 infants who showed a PSD peak. Thus, we did not do further analysis examining the association between HAZ and individually defined peak FC for this cohort. For the 36-month-old cohort, the mean PSD peak frequency is 8.38 Hz (SD = .887, N = 103/118). The PSD peak frequency was moderately correlated with the FC peak frequency, r = .56, p < .001. Regression analysis revealed that the association between HAZ and FC identified with individual PSD peak was close to the significance level (β = -.189., p = .08).

Figure S1. *The pipeline for EEG FC analysis in the source space used in the current study.*


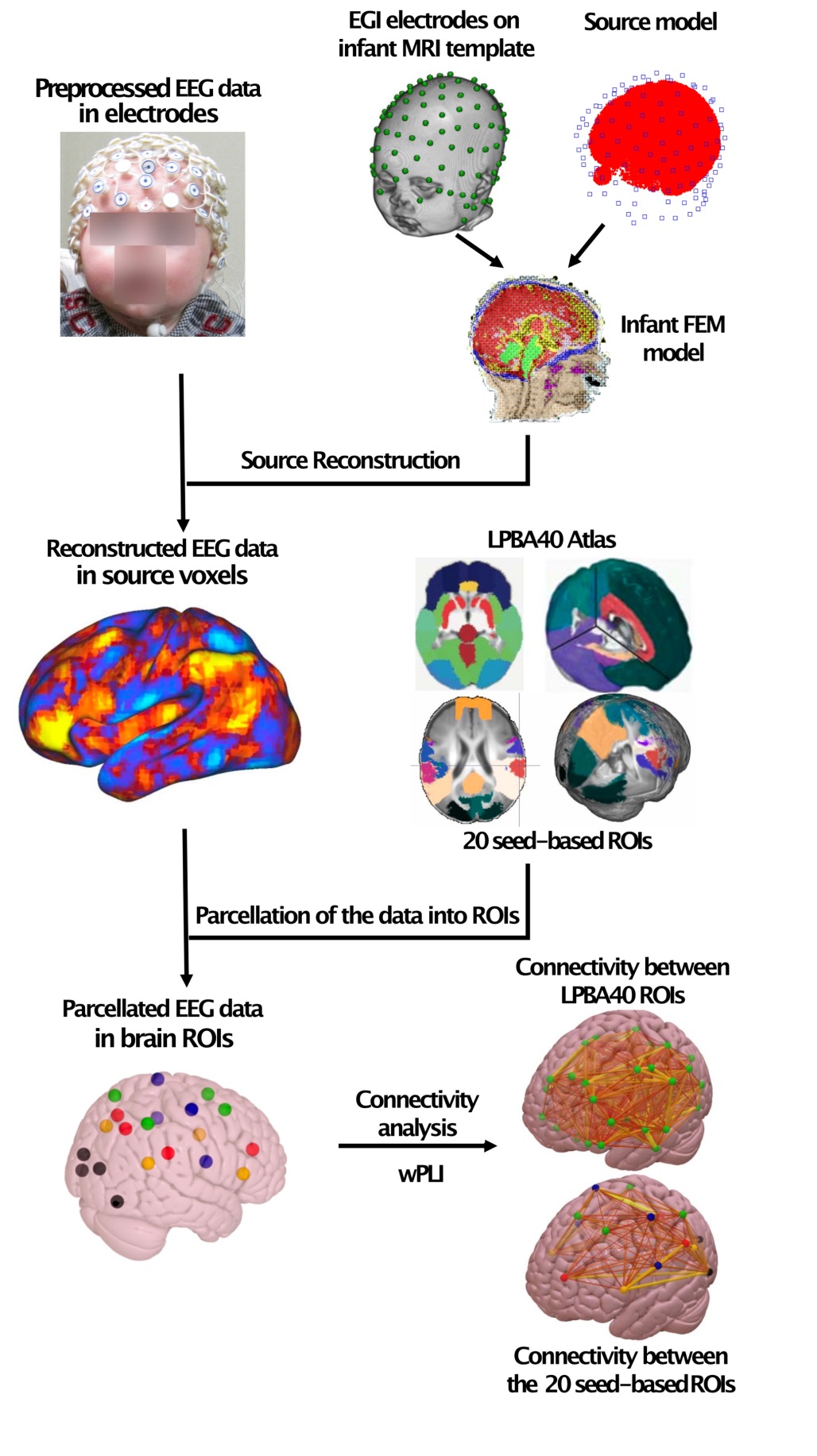


Figure S2. The IQ at 48 months for the stunted (white), middle HAZ (gray) and high HAZ (black) groups. Error bars stand for standard errors. ** *p* < .01 from the independent t-test between the stunted and high HAZ groups.

Figure S3. The average adjacency matrices for brain FC in the theta (top) and beta (bottom) bands for each 36-month-old group, i.e., the high HAZ, mid HAZ and stunted groups. The labels for the frontal, temporal, parietal and occipital ROIs are colored in blue, green, orange and red, respectively. These matrices correspond to the “brain FC plots” shown in Figure 4 of the main manuscript.

References in Supplemental Information

1. Delorme, A. and S. Makeig, *EEGLAB: an open source toolbox for analysis of single-trial EEG dynamics including independent component analysis.* Journal of Neuroscience Methods, 2004. **134**(1): p. 9-21.

2. Lopez-Calderon, J. and S.J. Luck, *ERPLAB: an open-source toolbox for the analysis of event related potentials.* Frontiers in Human Neuroscience, 2014. **8**.

3. Luyster, R.J., et al., *Neural measures of social attention across the first years of life: characterizing typical development and markers of autism risk.* Dev Cogn Neurosci, 2014. **8**: p. 131-43.

4. Righi, G., et al., *Infants' experience-dependent processing of male and female faces: insights from eye tracking and event-related potentials.* Dev Cogn Neurosci, 2014. **8**: p. 144-52.

5. Chaumon, M., D.V. Bishop, and N.A. Busch, *A practical guide to the selection of independent components of the electroencephalogram for artifact correction.* J Neurosci Methods, 2015. **250**: p. 47-63.

6. Mognon, A., et al., *ADJUST: An automatic EEG artifact detector based on the joint use of spatial and temporal features.* Psychophysiology, 2011. **48**(2): p. 229-240.

7. Xie, W., B.M. Mallin, and J.E. Richards, *Development of brain functional connectivity and its relation to infant sustained attention in the first year of life.* Dev Sci, 2018: p. e12703.

8. Oostenveld, R., et al., *FieldTrip: Open source software for advanced analysis of MEG, EEG, and invasive electrophysiological data.* Comput Intell Neurosci, 2011. **2011**: p. 156869.

9. Richards, J.E., et al., *A database of age-appropriate average MRI templates.* Neuroimage, 2016. **124**: p. 1254-9.

10. Richards, J.E.X., W., *Brains for all the ages: Structural neurodevelopment in infants and children from a life-span perspective*, in *Advances in Child Development and Behavior* J. Benson, Editor. 2015, Elsevier: Philadephia, PA. p. 1-52.

11. Hämäläinen, J.A., S. Ortiz-Mantilla, and A.A. Benasich, *Source localization of event-related potentials to pitch change mapped onto age-appropriate MRIs at 6 months of age.* Neuroimage, 2011. **54**(3): p. 1910-1918.

12. Pascual-Marqui, R.D., et al., *Assessing interactions in the brain with exact low-resolution electromagnetic tomography.* Philos Trans A Math Phys Eng Sci, 2011. **369**(1952): p. 3768-84.

13. Shattuck, D.W., et al., *Construction of a 3D probabilistic atlas of human cortical structures.* Neuroimage, 2008. **39**(3): p. 1064-80.

14. Hillebrand, A., et al., *Frequency-dependent functional connectivity within resting-state networks: An atlas-based MEG beamformer solution.* Neuroimage, 2012. **59**(4): p. 3909-3921.

15. Vinck, M., et al., *An improved index of phase-synchronization for electrophysiological data in the presence of volume-conduction, noise and sample-size bias.* Neuroimage, 2011. **55**(4): p. 1548-65.

16. Bathelt, J., et al., *Functional brain network organisation of children between 2 and 5 years derived from reconstructed activity of cortical sources of high-density EEG recordings.* Neuroimage, 2013. **82**: p. 595-604.

17. Mahjoory, K., et al., *Consistency of EEG source localization and connectivity estimates.* Neuroimage, 2017. **152**: p. 590-601.

18. Nolte, G., et al., *Identifying true brain interaction from EEG data using the imaginary part of coherency.* Clinical Neurophysiology, 2004. **115**(10): p. 2292-2307.
